# Obituary

**Published:** 1895-02

**Authors:** 


### Obituary.

Dr. M. C. Rowland died suddenly at his residence in Geneseo, N. Y., January 15, 1895, aged sixty-seven years. He had been in attendance upon the installation of the officers of A. A. Curtis Post, G. A. R., on Monday evening, January 14th, and returned to his home about 11 o'clock, when he retired for the night. Soon after midnight Mrs. Rowland was awakened by groans, but before medical aid could be procured her husband had expired.

Dr. Rowland was appointed assistant surgeon of the 61st Regiment, N. Y. V., September 2, 1862, was promoted to surgeon of the same regiment April 14, 1864, and was mustered out with his command July 14, 1865. The 61st Regiment was famous for its service, having first been commanded by Col., afterward Major General, Francis C. Barlow, and then by Col., now Major-General, Nelson A. Miles, United States army. During the winter of 1864 Dr. Rowland performed duty as an assistant in the field hospital of the first division, second army corps. After the war he located in Geneseo, where, by his courtly manners and professional attainments, he soon secured the confidence of the community as a medical practitioner of skill and a man of integrity.

He leaves a widow and two sons, who receive the sympathy of a large circle of friends in their sudden bereavement. The funeral was largely attended on January 18th, and the various civic and military societies, of which he was a member, attended in bodies.

---

Dr. Joseph H. Dunnigan, of Buffalo, died of diphtheria at the General Hospital on Friday morning, January 4, 1895. The disease was contracted while he was ministering to a patient suffering from diphtheria, and he was removed to the hospital, where he received every possible attention, under which, for a time, he seemed to improve. He, himself, did not believe that his malady would prove fatal, and in his opinion he is reported to have been sustained by his friends and attending physicians. A supply of diphtheria antitoxin, however, was obtained from New York and, meanwhile, the brave physician had grown worse. When it was suggested to him that he submit to the use of the antitoxin, he readily assented. He rallied a little at first, but died fifteen hours after its administration.

Dr. Dunnigan was born in Greenwood, Steuben county, this state, in 1864. His family lives near Rexville in the same county. After leaving the public schools in Greenwood, he attended the Canisteo Academy, and from there went to the medical department of the Niagara University, where he was graduated with honors. He was one of the staff at the Emergency Hospital, and later was house physician of the Sisters of Charity Hospital. He was a member of the Catholic Benevolent Legion, Ancient Order of Foresters of America and Ancient Order of Hibernians. He was attending physician to the three last named societies. He occupied an office at the corner of Smith and Elk streets, and had an extensive practice. He had been worn from hard work when he contracted the disease from one of his patients. A father and mother, a brother and two sisters survive the deceased. One of his sisters is Sister Cyril, of the Le Couteulx Deaf-mute Institute on Edward street.

His remains were taken to Rexville for interment.

---

Dr. Henry Goldthwaite, of New York, for the past sixteen years resident physician at the Fifth Avenue Hotel, and well known among its frequenters, died in his room at that house Thursday morning, January 3, 1895. He retired the night before apparently in usual health, but about 3 o'clock in the morning a ring came from his room, and a bell-boy found him in great suffering. His son, George, who was at the hotel, and Drs. Pease and Taylor were quickly summoned. He was conscious, however, only a few minutes and died very soon afterward of apoplexy.

Dr. Goldthwaite came of a well-known Southern family. He was born in Mobile on April 13, 1842. His father was Judge Henry Goldthwaithe, of the Alabama Superior Court. His great grandfather, William A. Graham, was governor of Alabama and secretary of the navy. Dr. Goldthwaite's mother was a Witherspoon. One of her ancestors, John Witherspoon, was a signer of the Declaration of Independence and president of Princeton College. The subject of this sketch was graduated from Princeton in the class of 1860. He entered the Confederate army as a private at the breaking out of the war of the rebellion. He was promoted again and again, and served on the staffs of Generals Ledbetter and Forney. He retired at the close of the war with the rank of major. In 1865, he became connected with the firm of Brown Brothers, cotton merchants, of Mobile. Later he studied medicine, and, coming to New York, he was graduated from Bellevue Hospital Medical College in 1876. For several years afterward he was an instructor at Bellevue. In 1878, he became resident physician of the Fifth Avenue Hotel. He was twice married, his first wife being a Miss Tarleton, of Mobile. There were two children by the first marriage. His second wife was Mrs. Caroline C. Munson, of Utica, whose daughter is the wife of Dr. Frank F. Ellenwood, of Attica, N. Y. The doctor was a member of the County Medical Society, the New York County Medical Association, the University Club and the New York Athletic Club. He was also a visiting physician to the City Hospital. The body was taken to his old home in Mobile.

---

Dr. David Worthington Miner, of Ware, Mass., died at his residence in that village on Wednesday evening, January 2, 1895, aged seventy-four years. Dr. Miner was one of the best known physicians in his region, and had continued the practice of medicine in the same office for nearly fifty years. He was a brother of the late Dr. Julius F. Miner, of Buffalo, a former editor of the Journal.

He was married in Northampton September 24, 1845, to Mary H., daughter of Joseph and Nancy Warner. Besides his widow he leaves four children, Mrs. Eliza N., wife of Prof. C. E. Garman, of Amherst; Affa S., wife of Prof. C. A. Tuttle, of Wabash College, Crawfordsville, Ind., and Miss Jean Miner and Dr. W. W. Miner, of Ware.

---

Dr. Alfred Lebbeus Loomis, of New York, died at his home, No. 19 West Thirty-fourth street, on Wednesday morning, January 23, 1895, aged 64 years. His death was caused by pneumonia and his illness was brief. Dr. Loomis was born in Bennington, Vt., was graduated from Union College in a class of '51, became a student of Dr. Willard Parker, and in 1853 received his degree as doctor of medicine from the College of Physicians and Surgeons. He spent two years as assistant physician on Ward's and Blackwell's islands, after which he established himself, in 1855, in the general practice of medicine in the City of New York. He cultivated the field of internal medicine early in his professional career and soon became authority on diseases of the chest. He has been physician to Bellevue Hospital and Mt. Sinai Hospital and for many years was consulting physician to Charity Hospital, on Blackwell's island. He was professor of medicine in the University of the City of New York for over thirty years. He served for two terms as president of the New York Academy of Medicine and has been president of the New York Pathological Society and the Medical Society of the State of New York. He was a liberal contributor to the medical literature of the day, some of his larger works being Lessons in Physical Diagnosis ; Diseases of the Respiratory Organs, Heart and Kidneys ; Lectures on Fevers ; Diseases of Old Age ; and A Text-book of Practical Medicine.

The curative properties of the air in the Adirondack region in cases of incipient tuberculosis, and its value as a prolonger of life in advanced cases, had an enthusiastic champion in Dr. Loomis, and he was active among those who opposed the extinction of the forests of this natural sanitarium. About twenty years ago it was reported that he went into the southern Adirondacks, where he was cured of lung trouble, after which he began to send his patients into that region.

Dr. Loomis was twice married and is survived by a widow and two children, the latter born of his first wife.

The funeral was held at the Church of the Incarnation, Madison avenue and Thirty-fourth street, on Saturday, January 26th, at 10 A. M.

